# Proteasome Inhibitors as a Possible Therapy for SARS-CoV-2

**DOI:** 10.3390/ijms21103622

**Published:** 2020-05-20

**Authors:** Lucia Longhitano, Daniele Tibullo, Cesarina Giallongo, Giacomo Lazzarino, Nicola Tartaglia, Sara Galimberti, Giovanni Li Volti, Giuseppe Alberto Palumbo, Arcangelo Liso

**Affiliations:** 1 Section of Biochemistry, Department of Biomedical and Biotechnological Sciences, University of Catania, 95123 Catania, Italy; lucia.longhitano@phd.unict.it (L.L.); d.tibullo@unict.it (D.T.); 2 Section of Haematology, Department of Scienze Mediche Chirurgiche e Tecnologie Avanzate “G.F. Ingrassia”, University of Catania, 95123 Catania, Italy; giuseppealberto.palumbo@gmail.com; 3 UniCamillus—Saint Camillus International University of Health Sciences, Via di Sant’Alessandro 8, 00131 Rome, Italy; giacomo.lazzarino@unicamillus.org; 4 Department of Medical and Surgical Sciences, University of Foggia, 71100 Foggia, Italy; nicola.tartaglia@unifg.it (N.T.); arcangelo.liso@unifg.it (A.L.); 5 Section of Hematology, Department of Clinical and Experimental Medicine, University of Pisa, 56121 Pisa, Italy; sara.galimberti@med.unipi.it

**Keywords:** SARS-CoV-2, proteasome inhibitors, endoplasmic stress, UPR response

## Abstract

The COVID-19 global pandemic is caused by SARS-CoV-2, and represents an urgent medical and social issue. Unfortunately, there is still not a single proven effective drug available, and therefore, current therapeutic guidelines recommend supportive care including oxygen administration and treatment with antibiotics. Recently, patients have been also treated with off-label therapies which comprise antiretrovirals, anti-inflammatory compounds, antiparasitic agents and plasma from convalescent patients, all with controversial results. The ubiquitin–proteasome system (UPS) is important for the maintenance of cellular homeostasis, and plays a pivotal role in viral replication processes. In this review, we discuss several aspects of the UPS and the effects of its inhibition with particular regard to the life cycle of the coronaviruses (CoVs). In fact, proteasome inhibition by various chemical compounds, such as MG132, epoxomycin and bortezomib, may reduce the virus entry into the eucariotic cell, the synthesis of RNA, and the subsequent protein expression necessary for CoVs. Importantly, since UPS inhibitors reduce the cytokine storm associated with various inflammatory conditions, it is reasonable to assume that they might be repurposed for SARS-CoV-2, thus providing an additional tool to counteract both virus replication as well as its most deleterious consequences triggered by abnormal immunological response.

## 1. Introduction

Since December 2019, infection with the severe acute respiratory coronavirus 2 (SARS-CoV-2) has become a worldwide emergency (pandemic) for which a rapid action is required [1,2]. In particular, COVID-19 (the illness caused by SARS-CoV-2) is overwhelming even well-organized national health care systems on a global scale [3,4]. Unfortunately, the symptoms of SARS-CoV-2 infection can vary in an unpredictable manner; there are asymptomatic cases as well as patients suffering from pneumonia, acute respiratory distress syndrome and multisystem organ failure [5,6]. Older patients and patients with preexisting respiratory or cardiovascular conditions appear to be at the greatest risk for severe complications and death [6,7]. In the absence of a proven effective therapy, current management consists of supportive care, including ventilation and treatment with antibiotics [8,9]. Moreover, patients are often treated with off-label therapies, including antiretrovirals, anti-inflammatory compounds, antiparasitic agents, and in a few cases, plasma from recently cured patients [10,11,12,13]. Antimalarial agents like chloroquine are used to block the virus entry, while new drugs like tocilizumab, anakinra or ruxolitinib [14], directed against a specific key element of the inflammatory response, are used to switch off the cytokine storm [15], as are antiviral drugs [16]. Nevertheless, in the absence of long-term and controlled clinical trials, there is no consensus on a “state of the art” therapeutic approach. Indeed, the use of drugs to stall the virus attack, followed by blocking viral replication and, in patients with signs of higher cytokine/chemokine release, the “pre-emptive” use of anti-IL6 or anti-IL1 blocking antibodies could be proposed [17,18]. Here, we review the potential role of proteasome inhibitors, based on previous studies showing that the ubiquitin–proteasome system is involved in the replication of a broad range of viruses.

### SARS-CoV-2

**Figure 1:**
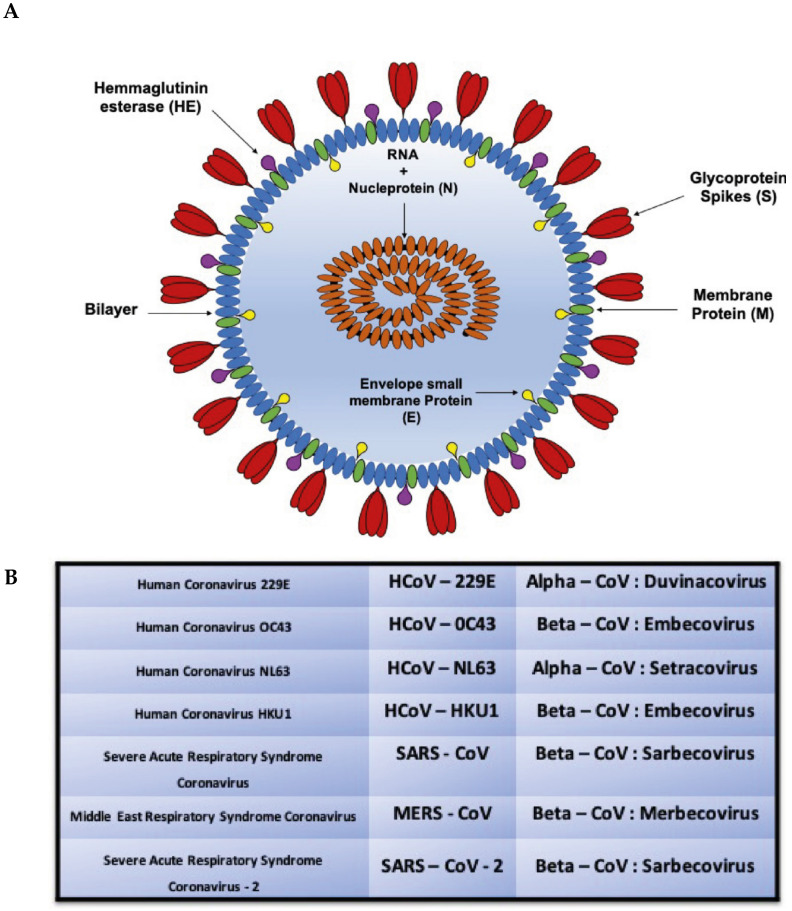
(**A**) Structure of the coronavirus. (**B**) The 7 Coronaviruses known to infect humans.

Coronaviruses belong to the Coronaviridae family in the order of Nidovales. They are approximately 65–125 nm in diameter and are single-stranded RNA viruses (+ ssRNA). The Coronavirus family includes four subgroups: α-, β-, γ- and δ-; among them, α- and β-CoV are capable of infecting mammals (Figure 1), while γ- and δ-CoVs mainly infect birds. Two well-known β-CoVs are SARS-CoV, responsible for the 2003 epidemic started in China (that caused 8000 infections and 800 deaths i.e., a 10% mortality rate), and MERS–CoV, which was responsible for the 2012 epidemic that began in Saudi Arabia (causing 2400 infections and 800 deaths i.e., a 35% mortality rate) [19,20]. Genomic analysis revealed that the new Coronavirus, SARS-CoV-2 is a β-Coronavirus. The SARS-Cov-2 viral genome is complex and resembles that of other coronaviruses. In particular, 75% of the genome is related to viral replicase genes from two open reading frames (ORFs), i.e., ORF1a and ORF1b, encoding for two polyproteins, pp1a (486 kDa) and pp1ab (790 kDa).The 1 ribo-some frame-shift occurs immediately upstream of the ORF1a stop codon, which allows the continuous translation of ORF1b to occur, producing a large polypeptide (pp1ab, 740–810 kDa) which is divided into 15 nsps. Proteolytic cleavage is mediated by the viral proteases nsp3 and nsp5 which, respectively, host a papain-like placenta domain and a 3C-like protease domain. Moreover, at short motifs called transcription-regulatory sequences (TRSs) that are located immediately adjacent to ORFs, the protease domain contains a conserved 6–7 nt core sequence (CS) surrounded by variable sequences [21]. Coronaviruses exhibit a round morphology and are constituted of several components, such as glycoprotein S (Spike), organized in trimers on the external part of the virion resembling a crown, from which they take their name; this protein determines the virus specificity for epithelial cells. In fact, it has been suggested that SARS-CoV-2 enters the host cell through the angiotensin-2 conversion receptor, ACE2, and CD26 (like SARS-CoV), expressed at both pulmonary and gastrointestinal levels (that’s why there are also symptoms at these compartments) [22]. Protein M crosses the envelope and interacts within the virion with the RNA protein complex. HE protein, the hemagglutinin esterase, is important during the virus release phase inside the host cell. The Protein E helps protein S attach to the target cells membranes. Finally, RNA gives rise to 7 viral proteins and is associated with the N protein, which increases its stability (Figure 1).

According to the evolutionary tree, SARS-CoV-2 is located close to the SARS-coronavirus group [23,24]. Recent studies have indicated significant variations in SARS-CoV and SARS-CoV-2, such as the absence of protein 8a and the fluctuation in the number of amino acids in protein 8b and 3c in SARS-CoV-2 [6]. Spike glycoprotein from the Wuhan coronavirus was modified by homologous recombination. Particularly, the spike glycoprotein of SARS-CoV-2 is a mixture of SARS-CoV bat and an unknown Beta-CoV [24]. The SARS-CoV-2 genome is 29.9 kb [25], while those of SARS-CoV and MERS-CoV are 27.9 and 30.1 kb, respectively [26]. The glycoprotein S comprises two subunits, S1 and S2 [27]. S1 determines the viral host flow rate and cell tropism with the key function domain–receptor-binding domain (RBD), while S2 mediates cell–cell membrane fusion through heptad repeat 1 (HR1) and 2 (HR2) [28,29]. Starting from the viral RNA, the synthesis of two 1a/1ab polyproteins (pp1a pp1ab) takes place in the host [30]. Transcription works through the replication–transcription complex (RTC), organized in double membrane vesicles, which continuously replicates and synthesizes subgenomic RNAs [31] and, in turn, encodes accessory and structural proteins. The endoplasmic reticulum (ER) and Golgi [32], genomic RNA, N protein and envelope glycoproteins come together to form viral bud particles. The vesicles containing virions then merge with the plasma membrane and release the virus. Transcription termination occurs in regulatory transcription sequences, located between open reading frames (ORFs), i.e., models for the production of subgenomic mRNA. In CoV, there are about 6 ORFs, and a frame-shift between ORF1a and ORF1b drives the production of pp1a and pp1ab, while the other ORFs encode for structural proteins S, E, M, N [30] and accessory proteins that interfere with the host’s innate immune response [2]. As mentioned, it has been suggested that SARS-CoV-2, like SARS-CoV, uses the ACE2 receptor to enter the host cell [33], but a N501T mutation in protein S of SARS-CoV-2 may result in a better binding affinity for ACE2 [34]. Systematic detection of β-CoV receptors has shown that human cells expressing ACE2 have improved SARS-CoV-2 access [35].

Angeletti et al. compared the gene sequence of SARSars-Cov-2 with that of SARSars-CoV. They analyzed the transmembrane helical segments in coded ORF1ab 2 (nsp2) and nsp3 and found that position 723 has a residue of serine instead of glycine, while position 1010 is occupied by proline instead of isoleucine [36]. The issue of viral mutations is the key to explaining potential relapses of the disease. At the protein level, no amino acid substitutions occur in rotavirus nonstructural protein families (NSPs), NSP7, NSP13, envelope, matrix or accessory proteins p6 and 8b, with the exception of NSP2, NSP3, spike protein, subdomain below, or RBD. Other recent research [37] has suggested that the mutation in NSP2 and NSP3 has a role in the infectious ability and differentiation mechanism of SARS-CoV-2.

## 2. Currently-Available Therapies

To date, there is no vaccine available, nor a specific antiviral therapy for SARS-CoV-2. The current therapeutic strategies used to cope with this pandemic are mainly based on symptomatic and respiratory support. On 28 January 2020, the WHO published a document summarizing its guidelines and scientific evidence derived from the treatment of previous HCoV epidemics [30]. Furthermore, the scientific world works constantly and is looking for an effective therapy, mainly based on strategies previously used against SARS-CoV and MERS - CoV. Interferons nebulization and various antivirals were initially used, including Nafamostat, Nnitazoxanide, Rribavirin, Penciclovir, Ffavipiravine, Rritonavir, Baricitinib, Lopinavir, Oseltamir and Arbidol. However, Remdesivir has been shown to be most effective in reducing viral load [38] (GS5734). As an RNA polymerase inhibitor, it has been shown, alone or in combination with chloroquine or interferon-β, to effectively block the replication of SARS-CoV-2 [39,40,41]. Based on experience in the fight against SARS-CoV and MERS-CoV, antiviral drugs and systemic treatment with corticosteroids commonly used previously in clinical practice, including neuraminidase inhibitors (Oseltamivir, Peramivir, Zanamivir, etc.), Ganciclovir, Aciclovir and Rribavirin, as well as methylprednisolone [42,43] for the influenza virus, are not valid for COVID-19 and are not recommended. Another effective drug is chloroquine (500 mg every 12 h) and hydroxychloroquine (200 mg every 12 h), drugs used for many years in the treatment of malaria, and today also in the treatment of rheumatoid arthritis. Several possible mechanisms have been studied. Chloroquine can inhibit the pH-dependent phases of the replication of different viruses [44], with a powerful effect on the infection and spread of SARS-CoV [45]. Furthermore, chloroquine has immuno-modulatory effects, suppressing the production/release of TNF-α and IL-6. Several studies have found that chloroquine interferes with the glycosylation of SARS-CoV cell receptors [45], and has worked in both the entry and postentry stages of COVID-19 infection in Vero E6 cells [41]. In Italy, a large survey conducted by the National Cancer Institute and the Pascale Foundation of Naples focused on the use of tolicizumab. It is a humanized IgG1 monoclonal antibody, directed against the IL-6 receptor and commonly used in the treatment of rheumatoid arthritis. Passive immunization has been successfully used for the treatment of infectious diseases, and 80 SARS patients were treated with convalescent plasma during the last major outbreak, while the same treatment did not occur for MERS. Since many patients have recovered despite the high number of deaths, this could also be a strategy for SARS-CoV-2.

## 3. Inhibition of the Ubiquitin–Proteasome System

The ubiquitin–proteasome system (UPS) is important for the maintenance of cellular homeostasis and also in viral replication processes. The UPS system is a complex process that leads to the degradation of ubiquitinated target proteins through the cutting action of the proteosome. Proteosome inhibitors are therefore molecules that are capable of inhibiting proteoasome activity, inducing the inhibition of the UPS response. We, in this review, focus our attention on the inhibition of proteasome activity by drugs already routinely used in many tumor pathologies. Some studies have focused on the role played by the ubiquitin–proteasome system and its inhibition in the process of entry and replication of the coronavirus [46]. Moreover, in the destabilization of antiviral proteins, viruses retain proviral or viral proteins by manipulating the ubiquitination processes through the expression of their own deubiquitination proteins (DUBs) [47], which have also been described for SARS-CoV [48,49]. On the other hand, the replication of numerous viruses also depends heavily on the activity of a functional UPS. Several studies have shown that virus infection leads to the accumulation of protein–ubiquitin conjugates, suggesting an important role of the increased ubiquitination process in ubiquitin–proteasome-mediated viral replication or protein degradation. Furthermore, the accumulation of proteins explained by the inhibition of proteasome activity causes a blockage of protein synthesis, endoplasmatic reticulum stress and cell death, leading to the inhibition of viral replication [50,51].

**Figure 2:**
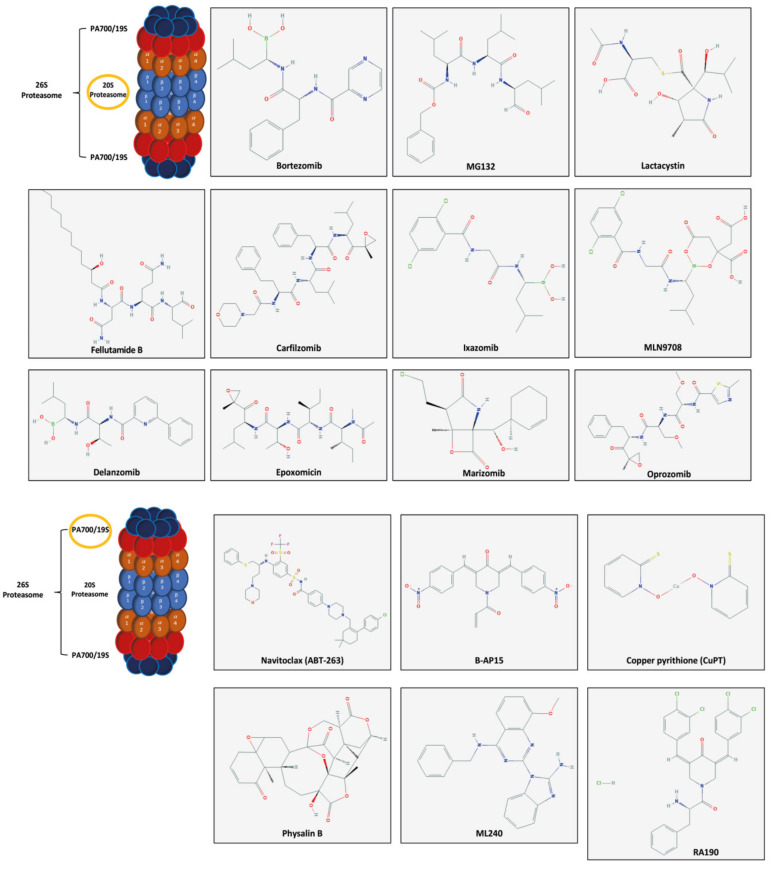
Chemical structures of several proteasome inhibitors. In yellow circle the subunit target of the drugs. Pubchem.

The best-known proteasome inhibitor is Bortezomib (Valcade) [50], which was approved in 2008 as a first-line drug in the treatment of multiple myeloma. Other inhibitors are MG132, Lactacistin, Carfilzomib, Ixazomib and others which are able to target the 20S and 19S subunits (Figure 2) [49,52]. Interestingly, Computational Drug Repurposing Studies showed that Carfilzomib is a good candidate for the treatment of COVID-19 [53].

As mentioned, one of the effects of coronavirus infection is the pro-inflammatory cytokine storm that occurs mainly in patients with severe respiratory conditions. The inhibition of the ubiquitin–proteasome system has proven effective in reducing the inflammatory response. In particular, a study by Moutzouris et al. [54] evaluated the anti-inflammatory effect of UPS inhibition in an airway smooth muscle (ASM) cell line. Specifically, this study demonstrated that cells treated with the MG132 inhibitor showed a reduction in IL-6 levels and other cytokines like sICAM-1, IP-10, MCP-1, MIF, RANTES, but also in the upregulation of MKP-1, a negative regulator of the serine/threonine protein kinases MAPK, which, once activated, plays a crucial role in a wide variety of cellular functions, ranging from proliferation to migration and synthesis of fibrotic and inflammatory proteins, including cytokines. Therefore, MAPK inhibition has emerged as an attractive strategy for reversing inflammation and remodeling in a wide variety of chronic inflammatory conditions [54]. Therefore, overall, the Moutzouris study suggests that UPS inhibition is an effective means of increasing the levels of the MAPK deactivator, MKP-1, and that, therefore, it could represent a possible therapeutic target in inflammatory conditions. Despite this, it is known that the ubiquitin–proteasome system is also important for the endocytosis and maturation of some viruses. Already in 2005, after the first SARS-CoV epidemic, in a study by Yi Yu et al. concerning the murine hepatitis virus (MHV-JHM strain, belonging to the Coronaviridae family), it was shown that the ubiquitin–proteasome system is involved in the release of the virus from the endosome to the cytosol during the virus entry phase. Proteasome inhibitors (lactacistin and MG132) inhibit MHV replication, demonstrating that the ubiquitin–proteasome system is involved in the early stages of virus replication, as the inhibitory effect was observed mainly from 0 to 6 h after treatment. It was stressed, however, that proteasome inhibition does not block the internalization of the virus. Furthermore, in the presence of MG132, most viruses remained in the vesicles, both endosomes and lysosomes, and therefore, are protected by RNase digestion [55]. Therefore, although MHV can be internalized in the cell in the absence of a functional ubiquitin–proteasome system, viruses within endosomes or lysosomes cannot be released into the cytosol [55]. Indeed, SARS-CoV, which binds to its functional receptor ACE2 [56], is internalized by endocytosis, and viral RNA is released from the endosome [56,57,58,59]. In the cytosol, the SARS-CoV genome is translated into two large polyproteins which are processed autocatalytically to produce nonstructural proteins (nsps), including all the nsps of the replicase viral complex [60,61,62]. Protected by double membrane vesicles (DMV), most likely originating from endoplasmic reticulum (ER) membranes [63], the genome is replicated and, by generating specific sets of subgenomic mRNA, structural and specific accessory SARS-CoV proteins are produced [61,62]. After assembly and budding processes in the endoplasmic reticulum of the Golgi intermediate compartment (ERGIC) [64,65], mature virions are released by exocytosis. Proteasome inhibition by various chemical compounds (e.g., MG132, Eepoxomycin and VelcadeBortezomib) seems not only to compromise the entry, but also the synthesis of RNA and the subsequent protein expression of different CoVs (e.g., Hepatitis Virus of mouse [MHV], feline infectious peritonitis virus, and severe acute respiratory syndrome CoV) [66]. A 2010 study by Raaben et al. showed that in cells treated with UPS chemical inhibitors, the synthesis of the viral CoV RNA and the subsequent protein expression were strongly reduced in all experimental conditions, while the entry of the virus was influenced only by chemical proteasome inhibitors [66]. In particular, the authors attempted to determine whether the inhibition of virus infection caused by MG132 was specific to this proteasome inhibitor or whether it was also observed when other proteasome inhibitors were used. Although MG132 has been shown to also inhibit proteases other than proteasome (i.e., cathepsin A and tripeptidyl peptidase II) [67], Eepoxomomine, Llactacystine and Velcade Bortezomib affect the proteasome more specifically [68,69,70]. Finally, a 2012 study showed conflicting data with previous work. Schneider et al. showed that the proteasomal inhibitor MG132 strongly inhibits the replication of SARS-CoV by interfering with the early stages of the viral life cycle, but other inhibitors (e.g., Lactacistin and Bbortezomib) only marginally affected viral replication, suggesting that the effect of MG132 is independent of proteasome inhibition, since MG132 also inhibits m–calpain. Interestingly, these authors observed that by using a calpain inhibitor (MDL28170), virus replication was inhibited, suggesting that MG132 does not inhibit SARS-CoV replication by ER stress induction, unexplained protein response or autophagy [47]. Since few studies to date have demonstrated the effects on coronavirus replication, we hypothesize that proteasomal inhibitors may negatively modulate virus replication, primarily acting on blocking protein synthesis due to an increase in stress on the plasma reticulum, and therefore, on the activation of the UPS. In the few papers published, bortezomib did not show great efficacy, but we have new inhibitors, some of which have the ability to bind the proteasome in an irreversible way, such as Ccarfilzomib. Interestingly, Wang et al. performed a molecular dynamics simulation followed by binding free energy calculations, demonstrating that Carfilzomib presented the best binding free energy, and therefore supporting the possible use of this proteasome inhibitor against COVID-19 [53].

## 4. Conclusions

**Figure 3:**
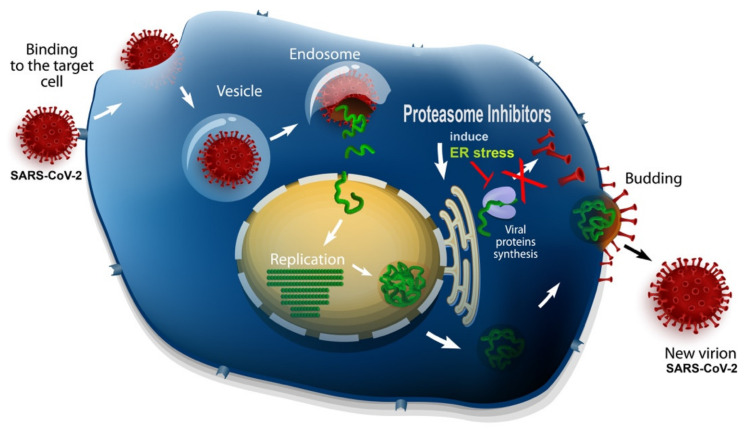
A schematic representation of the possible mechanism of proteasome inhibitors in virus replication.

In the absence of definitive protocols, effective antiviral care and a vaccine against SARS-CoV-2, the scientific community should explore possible therapeutic strategies, relying mainly on those used in previous epidemics. In this review, we summarized the current knowledge regarding the use of drugs, and in particular of ubiquitin–proteasome system inhibitors, which were proven to be powerful tools in reducing the cytokine storm and in inhibiting virus replication for other coronaviruses (Figure 3). Several molecules appear to be promising candidates; in particular, MG132, epoxomycin, bortezomib and carfilzomib have the potential to affect both viral replication as well as pneumonia and acute respiratory distress syndrome. Further studies are now warranted in order to establish the clinical efficacy of these compounds and to compare the clinical outcomes of patients.
